# Rapid and Label-Free Histopathology of Oral Lesions Using Deep Learning Applied to Optical and Infrared Spectroscopic Imaging Data

**DOI:** 10.3390/jpm14030304

**Published:** 2024-03-13

**Authors:** Matthew P. Confer, Kianoush Falahkheirkhah, Subin Surendran, Sumsum P. Sunny, Kevin Yeh, Yen-Ting Liu, Ishaan Sharma, Andres C. Orr, Isabella Lebovic, William J. Magner, Sandra Lynn Sigurdson, Alfredo Aguirre, Michael R. Markiewicz, Amritha Suresh, Wesley L. Hicks, Praveen Birur, Moni Abraham Kuriakose, Rohit Bhargava

**Affiliations:** 1 Beckman Institute for Advanced Science and Technology, University of Illinois Urbana Champaign, Urbana, IL 61820, USA; mpconfer@illinois.edu (M.P.C.); kf4@illinois.edu (K.F.); ytliu2@illinois.edu (Y.-T.L.); isharma3@illinois.edu (I.S.); aorr3@illinois.edu (A.C.O.); 2 Department of Chemical and Biomolecular Engineering, University of Illinois Urbana Champaign, Urbana, IL 61820, USA; 3 Head & Neck Surgery, Roswell Park Comprehensive Cancer Center, Buffalo, NY 14263, USA; subin.thenkunnelsurendran@roswellpark.org (S.S.); william.magner@roswellpark.org (W.J.M.); lynn.sigurdson@roswellpark.org (S.L.S.); mrm25@buffalo.edu (M.R.M.); amritha.suresh@ms-mf.org (A.S.);; 4 Head and Neck Surgery, Mazumdar Shaw Medical Foundation, Narayana Health City, Bangalore 560099, India; sumsum.sunny.dr@narayanahealth.org; 5 Department of Bioengineering, University of Illinois Urbana Champaign, Urbana, IL 61820, USA; lebovic2@illinois.edu; 6 Department of Electrical and Computer Engineering, University of Illinois Urbana Champaign, Urbana, IL 61820, USA; 7 Oral Diagnostic Sciences, University at Buffalo School of Dental Medicine, Buffalo, NY 14215, USA; aguirr@buffalo.edu; 8 Oral and Maxillofacial Surgery, University at Buffalo School of Dental Medicine, Buffalo, NY 14215, USA; 9 KLE Society Institute of Dental Sciences, Bangalore 560099, India; praveen.birur@gmail.com; 10 Karkinos Healthcare, Kochi 682017, India; 11 Department of Chemistry, University of Illinois Urbana Champaign, Urbana, IL 61820, USA; 12 Department of Mechanical Science and Engineering, University of Illinois Urbana Champaign, Urbana, IL 61820, USA; 13 Cancer Center at Illinois, University of Illinois Urbana Champaign, Urbana, IL 61820, USA

**Keywords:** discrete frequency infrared microscopy, precancerous condition, oral potentially malignant lesions, multimodal imaging, deep learning

## Abstract

Oral potentially malignant disorders (OPMDs) are precursors to over 80% of oral cancers. Hematoxylin and eosin (H&E) staining, followed by pathologist interpretation of tissue and cellular morphology, is the current gold standard for diagnosis. However, this method is qualitative, can result in errors during the multi-step diagnostic process, and results may have significant inter-observer variability. Chemical imaging (CI) offers a promising alternative, wherein label-free imaging is used to record both the morphology and the composition of tissue and artificial intelligence (AI) is used to objectively assign histologic information. Here, we employ quantum cascade laser (QCL)-based discrete frequency infrared (DFIR) chemical imaging to record data from oral tissues. In this proof-of-concept study, we focused on achieving tissue segmentation into three classes (connective tissue, dysplastic epithelium, and normal epithelium) using a convolutional neural network (CNN) applied to three bands of label-free DFIR data with paired darkfield visible imaging. Using pathologist-annotated H&E images as the ground truth, we demonstrate results that are 94.5% accurate with the ground truth using combined information from IR and darkfield microscopy in a deep learning framework. This chemical-imaging-based workflow for OPMD classification has the potential to enhance the efficiency and accuracy of clinical oral precancer diagnosis.

## 1. Introduction

Oral potentially malignant disorders (OPMDs) are precursors of over 80% of oral cancers [1] with reported malignant transformation rates ranging from 3 to 34%, correlating with the grade of dysplasia and prognostic biomarkers [2]. Though many OPMD cases will not progress to cancer, a large number of patients must still be screened for early detection of possible malignancy. The primary method for OPMD screening characterization is histopathology. The pathology of these oral cavity lesions may progress from dysplasia to invasive cancer following malignant transformation [3]. Over time, multiple OPMD histopathological grading systems have been proposed including five- [4] and three- [5] tiered scales proposed by the World Health Organization in 2005 and 2017, respectively, and a binary system [6], the latter included in the most recent 2022 WHO classification [7]. A second review was shown to increase interobserver agreement, but this may not be feasible in many laboratories [8]. A consensus report on the management of OPMDs underscored the need for histologic validation of the clinical diagnosis, as relying solely on clinical features can be misleading [9]. Part of the lack of confidence arises from current workflows that involve staining biopsy samples with hematoxylin and eosin (H&E). Routine tissue processing is a multi-step process yielding H&E-stained slides; quality may vary in different laboratories. In addition, interobserver variability is well documented in OPMD lesion diagnosis and grading [10]. Numerous prognostic markers using immunohistochemistry (IHC) have been suggested for OPMDs [11]; however, their development has been impeded by lack of a well-validated prognostic biomarker [11]. Widespread implementation of IHC staining for OPMDs is limited by reagent cost compared to conventional stains and expertise needed for interpretation. Innovations in digital dentistry have ranged from the use of algorithms to analyze stained images [12] to non-pathological advancements in intraoral scanners as adjuncts for physical impressions [13] and computational surgical planning [14]. Advancements in label-free imaging techniques offer the possibility of avoiding the need for staining in histological diagnoses and a potential alternative to current methods.

In this study, we examined the use of label-free infrared (IR) spectroscopic imaging to quantify spatial variations of vibrational modes in oral tissue, which can then be related to histological classifications through deep learning. IR imaging is a powerful tool for studying the spatial variation of the biochemical and molecular structure of tissues without the need for external dyes or reagents that detect molecular patterns [15,16]. The bulk of prior IR spectroscopic imaging data, including for oral cancer [17,18,19], has previously been acquired using Fourier transform infrared (FT-IR) microscopy that provides full IR spectral data for all pixels imaged [20,21,22,23,24,25,26,27,28]. Although FT-IR microscopy provides high-dimensional data due to the large spectral bandwidth, a large portion of this bandwidth does not contain biologically relevant vibrations; for example, the “cell-silent” region from ~1900 to 2700 cm^−^^1^ is devoid of biochemical features and increasing the number of spectral features for histopathologic classification is well-known to provide diminishing returns [29]. Many of the most biologically relevant vibrations, such as those associated with DNA, collagen, and other proteins, occur in the “fingerprint” spectral region, ~900–1800 cm^−^^1^ [15,16], presenting the strongest and most detailed optical signal reflective of molecular composition. As opposed to FT-IR imaging, using mid-IR tunable quantum cascade lasers (QCLs) has enabled discrete frequency infrared imaging (DFIR) [30,31] as a promising technique for biomedical imaging. By focusing on a select few bands in the fingerprint spectral region, QCL-based DFIR microscopes facilitate high-throughput IR imaging of samples relevant to histology, presenting an approach that is more time-efficient and data-optimized compared to FT-IR microscopy and can provide greater information than H&E-stained tissue alone.

One of the primary disadvantages of IR imaging is the relatively large diffraction limited spot size of IR light compared to visible light. The diffraction limited spot size of IR light limits incorporation of fine spatial and spatially variate spectral features, which are important for deep learning for histological applications [32]. Although IR imaging with a resolution finer than the diffraction limit can be achieved by using complementary modalities to probe IR response, for example, photothermal optical microscopy [33,34,35,36,37] or atomic force microscopy (AFM)-IR [38,39,40,41,42], these techniques are considerably slower than direct absorption DFIR. Moreover, AFM-IR provides a much finer spatial resolution than is required for this use case while presenting significantly more challenging sample preparation.

Here, we consider the combination of IR microscopy and optical microscopy to address the simultaneous challenges of obtaining high-resolution and rapid imaging data that retains chemical specificity and is scalable to clinical application for human biopsies. In particular, darkfield microscopy allows for unstained tissue sections to be imaged rapidly at the standard visible microscopy resolution. Darkfield microscopy is ideally suited for unstained tissue samples because it relies on the light scattered by the highly textured tissue sections to create image contrast. Brightfield microscopy, the standard method for stained tissue sections, is ineffective for unstained tissue as too much light is absorbed across the entire spectrum, resulting in low contrast. Given its capacity to rapidly generate high-resolution morphological images without the need for staining, darkfield microscopy stands out as an ideal companion to DFIR microscopy, especially when integrated into deep learning models.

Machine learning, specifically the sub-field of deep learning (DL), has greatly advanced in applications to pathology by leveraging artificial neural networks designed to mimic the human brain’s processing patterns. DL analyzes vast datasets, learns intricate patterns, and produces solutions autonomously [43] with a transformative impact increasingly evident across various domains, especially in machine vision. For imagery, it has refined techniques in segmentation [44], classification [45], and generation [46]. DL is also making major advancements in medical sciences such as models in pathology that aid in discerning histological components, forecasting disease prognosis and patient survival [47], enabling virtual staining [48], and generating synthesized histologic images [49]. These capabilities not only highlight intricate biological processes that may not be fully understood but also bridge the divide between raw data and insightful diagnostics. Progress has been made utilizing infrared imaging, both FT-IR and DFIR, for the development of DL models, showcasing the profound synergy between the two domains [50,51]. The models generated in this manuscript advance the current DL motifs for IR imaging by incorporating darkfield microscopy of unstained tissue as a low-cost and time-efficient secondary imaging modality. The current literature does not contain a method for rapid, label-free histopathological analysis of OPMDs. The objective of this manuscript is to describe a deep learning model based upon multimodal DFIR and darkfield microscopy to assist with label-free histopathological screening of OPMDs. The null hypotheses tested in this manuscript are that the tissue classes of non-epithelial, dysplastic epithelium, and non-dysplastic epithelium cannot be segmented using label-free infrared microscopy, darkfield visible microscopy, or a combination of the two methodologies.

## 2. Materials and Methods

### 2.1. Sample Preparation and Data Acquisition

Formalin-fixed paraffin-embedded (FFPE) human oral potentially malignant punch biopsies (n = 23) from Roswell Park Comprehensive Cancer Center (RPCCC) were used for this work. The biopsies were classified either as low-risk dysplastic (n = 5) or high-risk dysplastic (n = 18) lesions. Each tissue was sectioned onto an IR reflective MirrIR low-E slide (Kevley Technologies, Chesterland, OH, USA) at a 5 µm thickness. Slides were then deparaffinized by soaking in hexane (>98.5%, Fisher Chemical, Waltham, MA, USA) for 24 h prior to imaging. Adjacent sections were H&E stained for reference during histological annotation.

IR imaging was performed in a transflection geometry with a custom point scanning confocal DFIR microscope [30,52] that uses a QCL (Block Engineering, Southborough, MA, USA), a thermoelectric cooled mercury cadmium telluride (MCT) point detector (Vigo, Ożarów Mazowiecki, Poland), and a 0.71 N.A. refractive objective (Thorlabs, Newton, NJ, USA). IR images were acquired at 1238, 1546, and 1658 cm^−1^ at a magnification of 2 µm/pixel. Darkfield (DF) visible images were acquired on the DFIR system with a 30° illumination ring light and a 10x 0.30 N.A. objective (MPLFLN10x Olympus, Tokyo, Japan) illuminating a CMOS camera (BFS-U3–123S6C-C, FLIR, Wilsonville, OR, USA). The DFIR images were aligned to each other and processed using MATLAB 2021a (Mathworks, Natick, MA, USA) and the resulting DFIR and DF images were up-sampled or down-sampled, respectively, to 1 µm square pixels to allow for image alignment and dataset merging.

### 2.2. Dataset

From the overall sample size of 23 biopsies, 20 sections were used for training and 3 sections were used for validation and blind testing. This dataset division provided 2241 training patches and 320 testing patches. Patches were 256 × 256 pixels and were non-overlapping, and at least 50% of the pixels were tissue. Each image was annotated for 3 classes: (1) non-epithelium (connective tissue); (2) dysplastic epithelium; and (3) non-dysplastic epithelium under the guidance of a board-certified pathologist. The training dataset was composed of 87,592,748, 8,418,018, and 22,538,617 pixels of non-epithelium, dysplastic epithelium, and non-dysplastic epithelium classes, respectively. The testing dataset was composed of 16,239,690, 1,035,792, and 2,938,829 pixels of non-epithelium, dysplastic epithelium, and non-dysplastic epithelium classes, respectively.

### 2.3. Model Design

This model utilized the Fully Convolutional Network (FCN) architecture [53] with a ResNet50 backbone [54]. A ResNet50 backbone was selected as it has demonstrated good performance for semantic segmentation tasks. Training utilized the Adam optimizer [55], with a learning rate of 2 × 10^−4^ and a weight decay of 1 × 10^−5^. A cross-entropy loss function, which is well-suited for classification tasks, was used. A learning rate scheduler with a step size of 5 epochs and a decay rate (γ) of 0.5 was used to assist with model training. In order to minimize overfitting, an early stopping approach was integrated into model training. We have also included data augmentation, which involves applying random affine transformations to each patch in every iteration. To assess the robustness and consistency of the models, three parallel training experiments on identical datasets were performed, allowing for both the mean and standard deviation of the accuracy to be calculated. The framework was implemented in PyTorch 1.3, CUDA 10.1, and Python 3.7.1. Computations were performed on a single NVIDIA GeForce RTX 2080 SUPER GPU and Intel Xeon Silver 4216 CPU @ 2.10 GHz.

## 3. Results and Discussion

**Figure 1:**
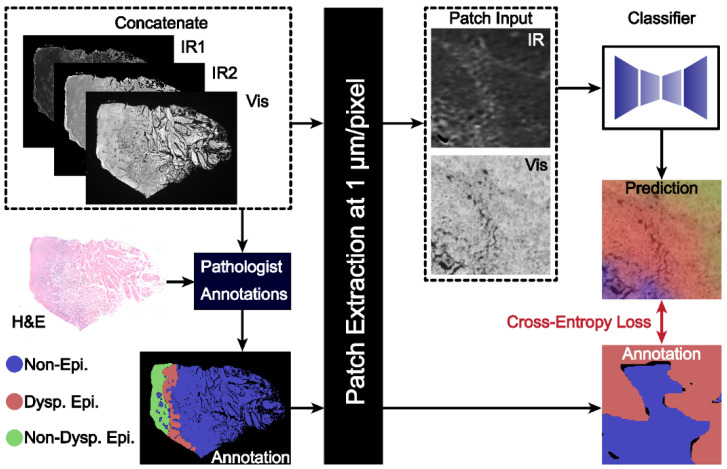
A combined IR and darkfield microscopy workflow for histopathology of oral potentially malignant tissues. The workflow combines the IR and DF images with pathologist annotations for the training dataset. Patches of images are passed to the classifier model to generate the final segmented image.

**Figure 2:**
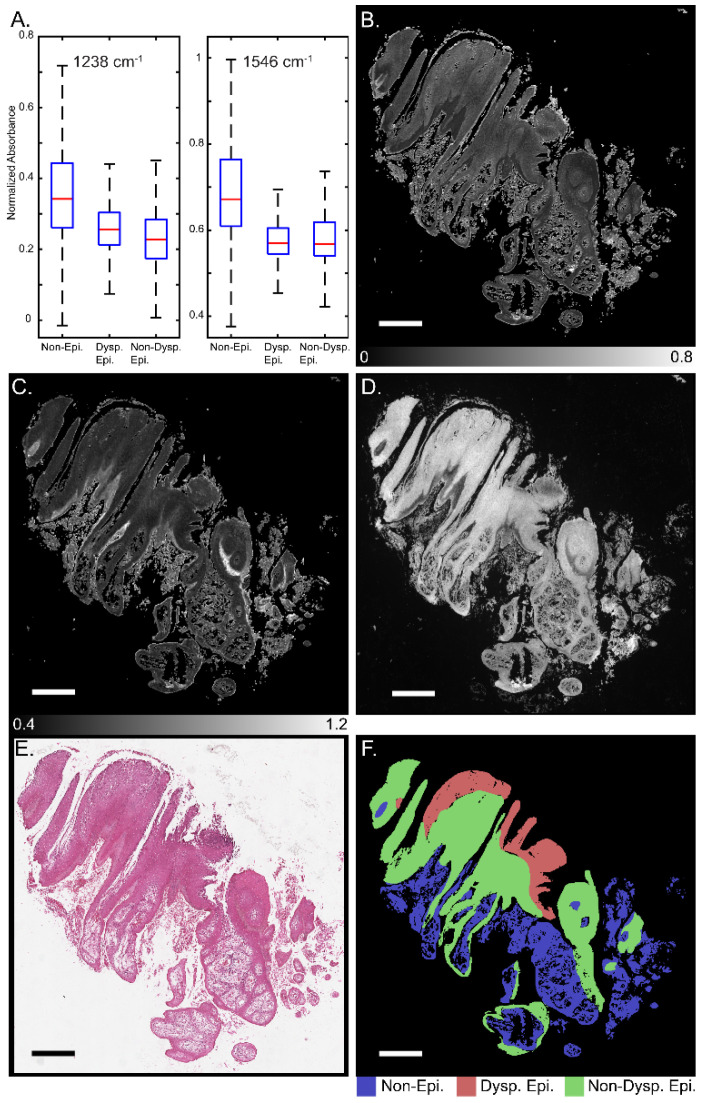
Discrete frequency IR imaging absorbance variation across oral tissue. (**A**) Normalized intensity distribution with outliers removed for 3 classes for absorbance at 1238 cm^−^ (left) and 1546 cm^−1^ (right). Representative whole biopsy IR images at (**B**) 1238 cm^−1^ and (**C**) 1546 cm^−1^. (**D**) Darkfield visible image of whole unstained biopsy section. (**E**) H&E-stained image of section adjacent to darkfield and IR imaged section. (**F**) class annotations for reference. Scale bar: 500 µm.

Figure 1 presents a schematic for segmentation of oral potentially malignant biopsies through a combination of IR and darkfield visible imaging. Overall, three discrete frequency IR images (1238, 1546, and 1658 cm^−^^1^) and a darkfield visible image of unstained tissue biopsies were acquired. The 1238 and 1546 cm^−1^ IR images were normalized to the 1658 cm^−^^1^ image to account for tissue thickness and density variations resulting in two final IR channels. These images were registered and, referencing an adjacent H&E-stained slide, were annotated for three classes: (1) non-epithelium (connective tissue); (2) dysplastic epithelium; and (3) non-dysplastic epithelium with pathologist guidance. This three-channel combined dataset and the registered annotations were used to train the deep learning classification model generated in this study. The IR bands used in this study, 1238, 1546, and 1658 cm^−^^1^, were chosen based upon known biologically relevant vibrational modes. The 1238 cm^−^^1^ mode is commonly assigned to the asymmetric PO2- stretch that commonly occurs in DNA and the Amide III mode from coupled N-H bending and C-N stretching [56,57,58,59,60]. The 1546 cm^−^^1^ mode is assigned to the Amide II band, a mixture of C-N stretching and H-N-C bending [59,60,61,62]. The 1658 cm^−^^1^ mode is part of the Amide I band, which is primarily attributed to C=O stretching that is indicative of protein secondary structure. The 1658 cm^−^^1^ band is commonly assigned to the alpha helix and random coil protein secondary structures and is generally the most intense component of the broader Amide I band. Amide I-normalized 1238 and 1546 cm^−^^1^ absorbance for the three different classes is presented in Figure 2A. Both the 1238 cm^−^^1^ and 1546 cm^−^^1^ bands provide median or distribution differentiation between all three classes. The amide I normalized absorbance for the non-epithelium class at both 1238 and 1546 cm^−^^1^ is greater than either the dysplastic or non-dysplastic epithelium classes. Absorbance at 1238 cm^−^^1^ is increased for dysplastic epithelium over non-dysplastic epithelium and the interquartile range at 1546 cm^−^^1^ is increased for non-dysplastic epithelium over dysplastic epithelium. The spatial spectral variations of the absorbance at 1238 cm^−^^1^ and 1546 cm^−^^1^ are shown in Figure 2B and Figure 2C, respectively, with the same section darkfield image shown in Figure 2D, the adjacent section H&E stained image shown in Figure 2E, and the class annotations shown in Figure 2F for reference. Increased median normalized 1238 cm^−^^1^ absorbance for dysplastic regions is reasonable, as prior studies have shown DNA variation for dysplastic samples compared to control and cancerous tissues [63]. Dysplastic epithelium shows increased nuclear content, atypia and irregular mitotic figures, which explain the DNA variation and increased absorbance. The atypical cells are characterized by morphological changes in terms of size, shapes of nuclear and cellular architecture, which are leveraged by the pathologists for diagnosis [64]. Nuclear content is another feature that increases with disease progression [65]. Although the DFIR spectral images used in this manuscript do not spatially resolve the nuclei, careful inspection of the darkfield visible images suggests nuclear features. Spectral differences at 1238 cm^−^^1^ were used in the literature to discriminate between hyperplasia, epithelia dysplasia, and oral squamous cell carcinoma [18]. The prior, FTIR microscopy, study found that the average spectral intensity at 1240 cm^−^^1^ increased as diagnosis became more severe (hyperplasia < dysplasia < squamous cell carcinoma) [18]. The variation in Amide I-normalized Amide II absorbance between connective tissue, dysplastic, and non-dysplastic epithelium is caused by protein concentration and secondary structure changes. The wide spectral distribution for the non-epithelium class for both 1238 cm^−^^1^ and 1546 cm^−^^1^ is likely due to the numerous different subclasses encompassed by the non-epithelium label.

Imaging of a representative OPMD sample, as shown in Figure 1, with dimensions of 2.8 × 3.7 mm^2^ required 274 s (~4.5 min) per IR band and 30 s for the darkfield visible image acquisition. The total imaging time for this sample was ~14 min resulting in 83 s/mm^2^ (~1.4 min/mm^2^) for all three IR bands and the darkfield image. This speed is one of the fastest with which IR images can be acquired today and compares favorably with the time needed for traditional histologic analyses. The sample dimensions provided were calculated based upon the actual imaging dimensions found by the smallest horizontal or vertical rectangle that encapsulates the sample. Further time optimizations are possible on systems that allow for either orientation of a rectangular scan area on a non-cardinal axis or for irregular scan areas. The imaging time here is longer than that of a single brightfield image of a stained tissue but eliminates the time and cost required for staining and subsequent processing and provides information that is unattainable from H&E alone.

**Figure 3:**
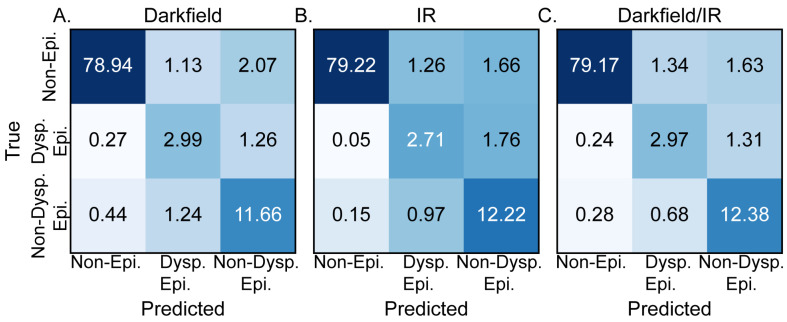
Comparison of test dataset confusion matrices, in percentage, for models trained on different imaging techniques. Confusion matrix for a model trained (**A**) exclusively using dark field images, (**B**) solely using IR images, and (**C**) by combining both IR and dark field images, showcasing the potential synergy between the two imaging methods.

DL was used to efficiently combine the observed variations in IR absorbance and darkfield microscopy for histopathological segmentation. The confusion matrices for the DL models trained using darkfield microcopy alone, DFIR alone, and the combination of darkfield and DFIR microscopy are shown in Figure 3A, Figure 3B and Figure 3C, respectively. Figure 3A, generated based upon darkfield microscopy alone, demonstrates an average accuracy of 93.6% and an F1 score of 0.815 (SD ± 0.021). These results indicate that, for OPMD segmentation, darkfield microscopy provides valuable diagnostic data, establishing itself as a dependable, relatively low cost, individual method for tissue segmentation. However, the accuracy may not be sufficiently high and specific for classification in a larger study or more sophisticated models. The model trained solely on IR images, Figure 3B, produced an overall accuracy of 94.2% and an F1 score of 0.799 (SD ± 0.016). The simultaneous increase in accuracy and decrease in F1 score for the IR only model compared to the darkfield only model is caused by increases in the accuracy for the non-epithelium and non-dysplastic epithelium classes but a decrease in the accuracy of dysplastic epithelium class prediction with increased confusion between dysplastic and non-dysplastic epithelium. The model performance differences based upon the training dataset indicate the different features that each method highlights that are useful for machine learning. The combination of the two imaging modalities, Figure 3C, provides equally good segmentation results. The combination of stain-free high morphological resolution and inherent chemical contrast provides an overall accuracy of 94.5% and an F1 score of 0.823 (SD ± 0.019). The deep learning metrics for three-class segmentation based upon DFIR alone, DF alone, and combined DFIR and DF support refuting the null hypotheses stated in the introduction and demonstrate that segmentation is achievable. While this combination of imaging techniques workflow shows good baseline performance, we anticipate that the chemical imaging data will become more important as we develop more complicated histology models or use microenvironmental data [66] for decision-making, which requires significantly more information. We note that the performance of these models should not be generalized to all acquisition parameters or tissues; each acquisition and analysis step should be considered part of a workflow that is self-contained. For example, using more spectral features may lead to an even higher accuracy. Here, we chose a few features to optimize speed of data acquisition and ensure that the time needed to record data could be short enough to be clinically relevant.

**Figure 4:**
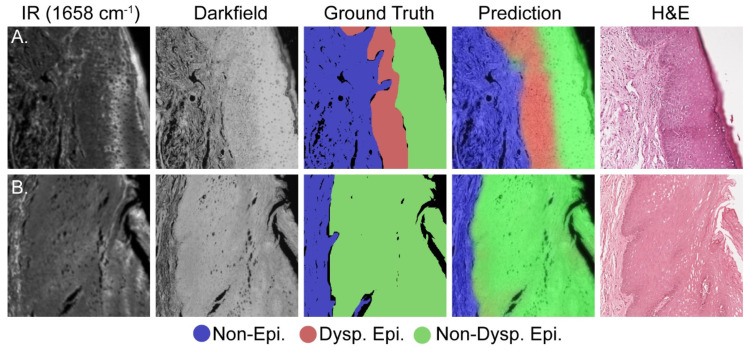
Comparative visualization of the deep learning model’s accuracy in identifying dysplasia. (**A**) Dysplastic sample; (**B**) non-dysplastic sample. Each set, moving from left to right, includes: an IR image, a dark field visible image, the ground truth annotation, the model’s prediction, and the adjacent H&E image.

Histology is an inherently visual field; therefore, projections of the darkfield/IR model onto the dataset are required to corroborate the quantitative results in Figure 3. Figure 4A presents a representative test set image containing all three classes whereas Figure 4B presents representative data containing only the non-epithelium and normal epithelium classes. Each row of Figure 4 presents, from left to right, the IR absorbance at 1658 cm^−^^1^, darkfield, combined model projection, ground truth, and adjacent section H&E-stained images to show the progression and agreement between the different data types. Although the IR/darkfield DL model test set projection images generally agree well with both the ground truth annotations and the adjacent section H&E images, there is some discordance at the borders of the classes. This discordance is best seen near the top middle of Figure 4A, where rete pegs of epithelium class protrude into the stroma, and in Figure 4B near the middle left, where the non-epithelium forms a peninsula in the epithelium (epithelial rete pegs). This slight, fine feature discordance is likely due to a combination of noise in the model, the use of the adjacent section H&E image to assist with annotations, and inherent individual to individual variation in annotations. Even noting these slight discordances between the test set projection images and the annotated ground truth, the model is still more than capable of identifying dysplasia and assisting pathologists. Here, we have just pointed out some features that can possibly result from noise and other variations as it is important to also point out imperfections in classification. However, in a larger study, the precise effects and their magnitude can be estimated but the increased number of samples can also lead to better fidelity of images.

The ability for the combined label-free three-band IR/darkfield deep learning model to identify dysplastic regions rapidly and accurately in OPMD biopsies points toward the ability to efficiently screen large populations in a cost-effective manner. The accurate and timely identification of dysplasia in OPMD biopsies is critical as the first step toward medical interventions, potentially improving both the efficiency and effectiveness of subsequent therapeutic strategies. Early detection will not only improve patient outcomes but should also allow for less invasive treatment as the disease was detected early in its progression. Historically, the medical community has relied heavily on traditional methods such as H&E staining and human expertise to interpret these results. Although these methods provide reasonable results, the development of artificial intelligence allows for computational assistance in diagnosis based upon patterns that are non-obvious and non-trivial for humans [67,68]. These findings, which highlight the potential of rapid label-free multi-modal imaging deep learning models for histopathological evaluations, are an example of artificial-intelligence-based histopathology.

The current model and dataset are a promising start for label-free multi-modal imaging-based segmentation of OPMD biopsies. The model presented here needs validation in a larger patient cohort, inclusion of variance in disease progression (i.e., no cancerous tissues were considered here), and samples from multiple institutions before this new model can be recommended for clinical application. Even with these stated limitations, the performance of the combined three-band DFIR/darkfield CNN model points toward the promise of this technique and lays the groundwork for future expansion to develop a clinically relevant model. Building on this foundation, another crucial area for exploration involves the integration of spatial information obtained through visible microscopy with the spectral data acquired from IR imaging. The current approach of simply concatenating these modalities appears to be suboptimal. Developing an architecture that is specifically tailored to leverage the unique aspects of these modalities could significantly improve the accuracy of the classification process. Such an improvement could, in turn, reduce the number of IR bands required, streamlining the data recording process. This efficiency gain is not just a technical improvement; it paves the way for the easier integration of this technology into clinical practice. By enhancing the model’s ability to accurately segment OPMD biopsies with less data and greater precision, the pathway to adopting this innovative diagnostic tool in clinics becomes more feasible, potentially transforming patient care in oncology and pathology departments. Future applications of this model can assist in the precise quantification of dysplastic epithelium of OPMDs into low-grade and high-grade categories, which may reduce diagnostic discordance and subjectivity.

## 4. Conclusions

This work demonstrates the ability for a chemical imaging workflow, based upon a simple three-discrete-frequency IR image dataset paired with darkfield microscopy as inputs to a DL framework, to classify oral potentially malignant lesions without the use of stains. IR absorbance at 1238 and 1546 cm^−^^1^, normalized to 1658 cm^−^^1^, provided the necessary chemical information, and darkfield visible microscopy contributed higher resolution morphological data than is available with standard IR microscopy. Deep learning models trained independently with either of these modalities fall short of the accuracy provided by the combination of IR and darkfield imaging, pointing to the utility of multimodal stainless imaging for histopathology. This work paves the way forward for high-throughput rapid screening of OPMDs by developing deep learning segmentation models based upon multi-modal label-free imaging to assist clinicians.

## Data Availability

Given the size of the data, it is available from the authors upon reasonable request.
